# Innovation in UK independent homecare services: A thematic narrative review

**DOI:** 10.1111/hsc.13954

**Published:** 2022-08-04

**Authors:** Diane Burns, Cate Goodlad, Kate Hamblin, Karla Zimpel‐Leal

**Affiliations:** ^1^ University of Sheffield Sheffield UK; ^2^ Oxford Brookes University Oxford UK

**Keywords:** independent homecare providers, Innovation, outcomes, social care policy

## Abstract

This paper reports the findings of a thematic narrative review of peer‐reviewed articles exploring innovation in UK independent homecare services published between January 2009–August 2021. Our analysis of 15 papers reveals four broad innovation types: personalised funding, operational models, workforce development and assistive technology. We conclude that research focused on innovation in independent homecare offers important insights into the positive and negative outcomes of different types of innovation for providers, care workers and people receiving care. There are, however, also areas which are neglected and need further elaboration, including more robust evidence of outcomes and clearer articulation of innovation processes.

**What is known about this topic**

- Innovation became a particular focus of UK policy from 2009 onwards, hailed as the solution to the crisis in adult social care more generally and in homecare provision specifically.
- There has been national and local investment to support innovation in homecare in the UK.
- Health and social care research has largely focused on the positive effects of innovation, neglecting to define the concept or explore its complexities.

**What this paper adds**

- We map the scope and focus of extant academic research into innovation in independent homecare organisations in the UK.
- The paper identifies four types of innovation in independent homecare organisations explored in the existing academic literature.
- We highlight gaps in knowledge and propose an agenda for future research.

## 1 INTRODUCTION

In the UK, the adult social care (ASC) sector has been characterised as being ‘in crisis’ due to decreased budgets and a mismatch between the demand for and supply of care. In response, innovation is a policy priority for ASC in the four UK nations, yet fragilities and fragmentation in the sector present significant barriers (Schröder & Howald, 2017). The UK has been argued to be in a period of ‘destatization’ (Cunningham, 2016) since the economic crash of 2008, changing the way ASC is funded, structured and delivered, leaving many long‐standing problems and persistent challenges unresolved, including high care workforce turnover and job vacancies (Hussein, 2017; SCIE [Social Care Institute for Excellence], 2019; Skills for Care, 2019).

Responsibility for ASC policy, legislation, standards and the allocation of funding is devolved to the four nations whereas the delivery of services is the responsibility of multiple localities (151 local authorities in England, 22 in Wales, 32 councils in Scotland and five health and social care trusts in Northern Ireland [Gray & Birrell, 2013]^i^). Homecare is a subsector of the wider ASC sector and whilst some localities have their own, ‘in‐house’ services, the majority of homecare is ‘contracted out’ to homecare organisations to provide these services, which we refer to herein as the ‘independent homecare sector’. The independent homecare sector is made up of for‐profit, non‐profit and third‐sector organisations, providing around 96% of the provision in England, 82% in Wales, 71% in Northern Ireland and 53% in Scotland (UKHCA, 2019). Some of these homecare organisations specifically target the private market (comprising ‘self‐funders’ who pay for their own care), whilst others deliver care commissioned by localities or operate a mixed economy, relying on both self‐funders and state‐funded care contracts. The market for independent homecare organisations is precarious with some providers ceasing operations, or handing contracts back to localities, citing squeezed margins and underfunding (ADASS, 2019; The King's Fund, 2018, 2019).

Given the significance of the independent homecare sector to the provision of care in the UK, it is important to understand the relationship between innovation in these organisations—hailed as a means to create stability and improve outcomes—and service quality. Innovation is a prominent focus of policy, practice and research, as will be explored in the next section, yet the wider literature is characterised by two issues: first, conceptual ambiguity with no widely accepted definition of ‘innovation’ (Crossan & Apaydin, 2010; Linton, 2009) and second, that the focus of much research is on identifying positive outcomes of innovation, thereby overlooking the specifics of how innovation is generated and developed, and why some innovations fail (Seelos & Mair, 2012, 2016).

This paper aims to provide insight into innovation in UK independent homecare organisations and services since 2009–10 (when innovation became a particular policy focus), identifying the types of innovation examined in academic research. Drawing on an understanding of the term ‘innovation’ as referring to the operationalisation of some kind of potential with a commercial or social motive by implementing new adaptive solutions that create value (e.g. Singh & Aggarwal, 2021), we start by briefly describing and critiquing UK policy context vis‐à‐vis innovation. We next introduce the methodology used to search, filter and review past research on innovation in independent homecare organisations. Following an inductive thematic analysis of the selected papers, four areas of innovation and their corresponding outcomes are identified. Finally, we discuss key areas where further elaboration is required.

## 2 INNOVATION, SOCIAL POLICY AND ASC

Although innovation has been a prominent discourse in UK parliamentary debates since the 1960s, it is a concept that lacks a precise definition (Perren & Sapsed, 2013). It tends to be understood as a policy agenda through which governments have sought to strengthen the UK's economic base (Edmiston, 2015; Sirovatka & Greve, 2016) and tackle societal challenges (e.g. UK Industrial Strategy 2017). The 2000 Green Paper, *A Quality Strategy for Social Care*, set out actions to improve the quality of social care services, promoting innovation as a key mechanism to do so whilst also stabilising the cost of care. To support the delivery of these aims, the then Government established the Social Care Institute for Excellence (SCIE) (which became an independent charity in 2002) to create a network of innovative providers, commissioners and interested citizens and encourage the spread and scaling up of localised innovations in social care (SCIE , 2019). There has also been funding available to the sector, such as the Social Impact Bond ‘trailblazers’ to support English local authorities (LAs) to encourage homecare providers to ‘rise to the challenge and to develop innovative and high‐quality care and support options’ (HM Government, 2012: p. 45–46).

Specific to innovation in ASC more broadly are two key policy priorities: (1) the ‘personalisation’ of care and (2) the deployment of technology to improve care quality and contain costs. ‘Personalisation’, whilst itself not well‐defined (Glasby & Littlechild, 2009; Needham, 2011), has been a central part of policy rhetoric, promoting core values of ‘choice’ and ‘control’ since the 2000s (Lymbery, 2014) but has been linked with ASC since the 1980s as part of the independent living movement. The *Community Care (Direct Payments) Act* (1996) gave localities the power to make direct payments^ii^ to people to control their own care, which was made a duty in England and Wales in 2001 and Scotland in 2003 (Hall et al., 2020; Needham, 2011). As such, over time ‘quality’ care has come to be understood as care that is personalised (amongst other features); in turn, personalised funding is a mechanism to deliver personalisation. In terms of investment, in England, the *Partnerships for Older People* pilots (2006/7 and 2007/8) were established to encourage innovation and quality through the use of direct payments and individual budgets^iii^ (Department of Health, 2006).

Technology in ASC has also been a key area of policy focus and public investment, often coupled—or at times treated as synonymous with—innovation. Policymakers have frequently pronounced technology's ‘transformational’ potential, with innovations in both the design of new products and systems or the application of existing devices to caring contexts cited as having the ability to increase care quality and workforce capacity whilst reducing costs (Hamblin, 2022). Investment has included the Care and Health Improvement Programme's Social Care Digital Innovation Programme and NHS Digital's Digital Social Care Demonstrator Programme, both of which provided funding for localities to develop digital ASC pilots with stakeholders, including care providers. Other opportunities available to homecare organisations as applicants (not only as partners) included Digital Social Care (DSC), Local Government Association (LGA) and Department for Health and Social Care (DHSC) and NHS Digital funding. Through these initiatives, emphasis has been placed on the development, adoption and diffusion of digital technologies within ASC and homecare (Wright, 2020).

## 3 CRITIQUES OF INNOVATION

Whilst government policy and initiatives assert innovation is good for the economy and society, some scholars argue that definitional ambiguity allows multiple understandings of the term to flourish in policy, practice and research (Crossan & Apaydin, 2010; Godin & Vinck, 2017). Scholars have also warned that research risks reproducing policy's pro‐innovation bias (Fagerberg et al., 2013; Godin & Vinck, 2017) and that it tends to be treated as an outcome, therefore implying ‘that innovation occurs when desired outcomes such as positive change can be observed’ (Seelos & Mair, 2012: p. 45).

Reviews of research investigating innovative practices and services in ASC in the UK more broadly than our focus on independent homecare organisations have found significant gaps in knowledge and data or weak evidence of positive outcomes. A Local Government Association (LGA) (2017) review of the use of procurement to encourage innovation found a problematic lack of data on innovative activities; other reviews of areas of innovation in ASC related to technologies (but not focused on independent homecare provision) have reported conflicting evidence of positive outcomes for different users (Davies et al., 2013). In reviews where positive outcomes of innovative practice in ASC were found, and innovation transfer to other localities was attempted, there was little acknowledgement of how localised cultural, social and economic contexts and condition‐mediated outcomes (Trivedi et al., 2013, focused on inter‐professional working) and others have highlighted a lack of evidence on specific factors that result in successful and sustainable innovations (Dawson et al., 2015; Trivedi et al., 2013; related to dementia care).

Whilst the policy discourse presents innovation as a means to ameliorate some of the effects of an under‐funded, under‐staffed and fragile system (DHSC [Department of Health and Social Care], 2017; SCIE , 2019), it has been argued that evidencing outcomes of innovation can be difficult to achieve when the agenda contains the dual, and often conflicting, objectives of containing costs *and* improving care quality. For example, personalisation has been highlighted as a mechanism to stimulate change in business processes, resulting in greater efficiencies including tightening access to services, greater auditing and increased use of IT systems (Carr, 2010) but this link to cost savings and efficiencies has been argued to be at odds with the drive to improve care quality (Pearson & Ridley, 2016).

Whilst these reviews illuminate the range of innovations more broadly within ASC and what factors support or hinder their effectiveness, less is known about innovations specific to the independent homecare subsector. In this paper, we report the first review (to our knowledge) of peer‐reviewed research on innovation in independent homecare organisations, asking: (1) What modes of innovation does research show to be emerging in the independent UK homecare sector? and (2) What are the reported outcomes of these innovations?

## 4 METHOD

We conducted a narrative summary review (Dixon‐Woods et al., 2005; Greenhalgh et al., 2018) of the literature focused on innovation in, or associated with, independent homecare organisations in the UK. Our aims were to provide further nuance to the conceptualisation of innovation by examining the variation in the type of innovations, their prevalence and outcomes. As innovation is a contested concept, a narrative review is appropriate as it combines interpretive and discursive methodologies to provide clarification and insight (Greenhalgh et al., 2018) and has the potential to allow theory‐building through thematic analysis (Hammersley, 1997). Through thematic analysis within the narrative review method has been critiqued as ‘limited in its ability to deal with contradictions, other than by describing them’ (Dixon‐Woods et al., 2004: 15), we argue that whilst highlighting contradictions may appear ‘descriptive’, it is crucial for building an understanding of how ‘innovation’—as a vehicle for tackling problems of fragility and poor quality care in the ASC that is also conceptually ambiguous – is defined and implemented. This approach brings studies using a range of research methods into the scope and allows us to examine a phenomenon that is ill‐defined, with the potential to generate new insights and develop hypotheses or aims for future fieldwork (Dixon‐Woods et al., 2005).

**FIGURE 1:**
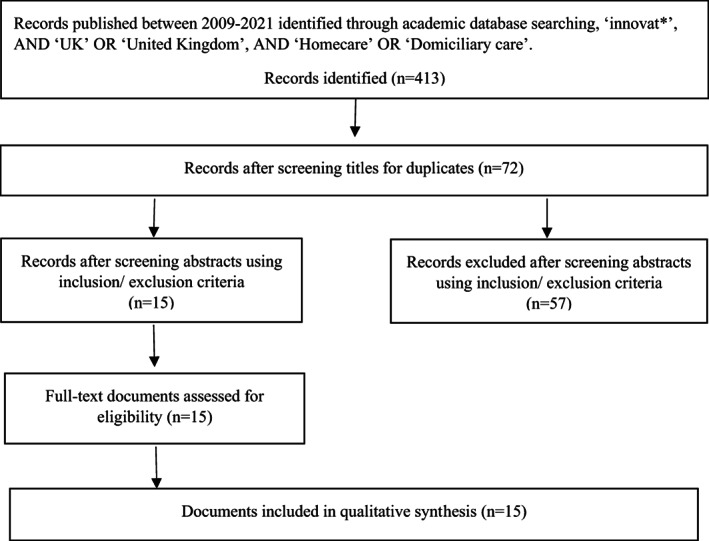
Search flow diagram to show the process of including and excluding documents

Six online databases (ASSIA, British periodicals, ProQuest, Social Science Premium Collection, MEDLINE, Scopus) were searched, using the following search terms: ‘innovat*’ (a ‘wild card’ search ‘innovat*’ allowing for the return of results which included ‘innovation’ and ‘innovative’), AND ‘UK’ OR ‘United Kingdom’, AND ‘Homecare’ OR ‘Domiciliary care’.^iv^ The searches took place in August 2021 and included English language literature published from January 2009, including international literature focused on innovation in the UK. The searches returned 413 academic papers, reduced to 72 after the removal of duplicates. The reference lists of each selected paper were also hand‐searched, but no additional papers were found. Two members of the research team independently read the abstracts of all 72 papers, applying inclusion and exclusion criteria, before discussing and agreeing on which papers to include for further analysis. The aims of the review informed the inclusion and exclusion criteria shown in Table 1 and the search flow diagram is summarised in Figure 1. Our approach did not include criteria related to methodology or paper quality—as is common in scoping reviews—because this is problematic when conducting a narrative review examining qualitative research as it would assume a unity of methods and would ignore the diversity of methodologies within qualitative approaches (Dixon‐Woods et al., 2004).

**TABLE 1:** Inclusion and exclusion criteria

Inclusion criteria	Exclusion criteria
Published between January 2009 and August 2021 Included a focus on innovation in ASC in the main text Included a focus on paid care delivered by independent homecare providers or commissioning of independent homecare (including live‐in care) Reported empirical research (all methodologies) Reported research included a focus on the UK	Published before January 2009 and after August 2021 No focus on innovation in ASC in the main text No focus on independent homecare provider organisations Articles focused on residential/ nursing long‐term care/ day care/ state‐provided homecare/ children's services Purely theoretical papers Wholly focused on innovation outside of the UK

*Note*: Key: ASC: Adult Social Care; UK: United Kingdom.

After filtering, 15 papers met our criteria (10 empirical research articles drawing primarily on qualitative research, three commentary papers and one review paper) (Table 2). We adopted a data‐driven approach to thematic analysis, where the themes were generated through the exploration of the literature (Dixon‐Woods et al., 2005). Our analytic approach involved several steps. Two members of the research team independently read each paper several times to identify the type of innovation. Together, the team members then compared their findings and grouped similar innovation types together. This resulted in four groupings, which we labelled to reflect the topics examined. Papers within each category were re‐analysed thematically to reflect the focus of the innovation in combination with the objectives driving its implementation. Where discernible, we identified the reported outcomes of the innovation for the people involved in giving and receiving care.

**TABLE 2:** Papers summarised by theme

Reference	Examines	Study type	Innovation focus & effect
*Personalised funding*			
Baxter et al. (2011)	Effects of implementing pPBs	Research interviews exploring risks/opportunities of introducing PBs & case studies of 4 homecare organisation pilots	Innovation: increased use of PBs Effect: difficulties around financial and workforce planning; few care workers left but often returned, resulting in additional recruitment costs
Sutcliffe et al. (2012)	Care arrangements & use of resources	2 national surveys of commissioners & interviews with commissioners in 20 Localities implementing PBs	Innovation: flexible use of PBs Effect: require Localities to be creative & flexible in their use of internally held budgets
Baxter et al. (2013)	Brokering method on competition & choice	Interviews with commissioners, support planners, homecare organisation managers & care recipients in 3 Localities	Innovation: increasing choice Effect: LAs found not to be able to guarantee choice
Brookes et al. (2015)	Research needed to assist LAs in developing services	Interviews/focus groups with 9 Localities; case studies of interventions in 3 Localities to implement the personalisation agenda	Innovation: integrating personalisation & care with housing Effect: renewed partnerships, culture shifts & engagement; challenges include achieving long‐term culture change
*Alternative operational models*			
Jones (2009)	Care outcomes of social enterprise companies	Commentary on setting up social enterprises	Innovation: flexibility and service delivery Effect: employee participation in decision‐making increased
Munoz (2013)	Process of co‐production in a rural setting & its effects	Participatory action research with health care professionals, community members & Locality commissioners to establish a social enterprise	Innovation: coproduction of social enterprise model Effects: empowerment & personal satisfaction but also pressure & frustration
Giraud et al. (2014)	How innovations in homecare organisations are implemented	Analysis of 3 homecare organisations in Germany, Scotland & Switzerland	Innovation: ‘bottom up’ generation of new services Effect: over time ethos shifted towards markets, measurement and income
Westwood (2016)	Diversification of provision, including homecare, to meet the needs of people identifying as LGBT	Review of extant literature and research studies	Innovation: human rights based‐provision Effect: consequences for recruiting a diverse workforce with a greater range of experiences
Glasby et al. (2018)	Care provider size, outcomes and costs	Interviews with stakeholders from different sized organisations	Innovation: relates to size/model of organisation Effect: micro‐providers (<5 paid/unpaid staff) deliver more personalised care, support staff autonomy & continuity
Cameron et al. (2019)	Effect of extra‐care housing on working conditions	Interviews with employees in 4 companies	Innovation: extra‐care housing model Effect: despite promoting responsiveness & flexibility, employees experienced workplace pressures found in conventional homecare settings
Evans et al. (2020)	Extra‐care housing for people living with dementia	Longitudinal project with residents and staff from four extra care housing schemes	Innovation: person‐centred care and support, flexible commissioning and staffing. Effects: appropriate design, suitable location of the scheme and delivering services that address these issues during a period of reduced public spending
*Training regulation catalysing change*			
Rainbird et al. (2011)	Links between regulation & training	Interviews with care organisations involved in skill development and case studies of 13 homecare organisations & training agencies	Focus: training regulation and quality assurance mechanisms Effect: provides impetus for innovation and building capacity in the sector (learning hubs), improves information (on the benefits of training & learning), increases staff motivation to pursue qualifications
*AT enhancing care*			
Roberts et al. (2015)	AT support for people with chronic pain	Case study of AT in rural area	Focus: use of AT Effect: provides opportunities to enhance care interactions but impeded by poor technological infrastructure
Doughty and Williams (2016)	Prescription, uptake & utilisation of AT	Commentary on the reasons for low uptake	Focus: use of AT Effect: low uptake linked to low levels of training, information & support
Caleb‐Solly (2016)	Assistive robotics potential to meet care needs	Commentary on the potential to support older people to remain independent	Focus: use of assistive robots (when integrated with smart home sensors & healthcare databases). Effect: speculative claims that AT can enhance the quality of life and independent living

Abbreviations: AT, Assistive Technology; PB, Personal Budget.

## 5 FINDINGS

Our analysis identified four narrative themes in the literature related to innovation and UK‐independent homecare organisations: (1) personalised funding and choice; (2) alternative operational models; (3) training regulation catalysing change; (4) assistive technologies for enhanced care. The aims, type and outcomes of innovations within each theme are summarised in Table 2.

### 5.1 Personalised funding and choice

Papers from this category conceptualised personalised funding as a mechanism through which innovation was operationalised, particularly in driving change to enable people to determine and shape the care they receive and who provides it. These papers focused on if and how the implementation of personal budgets could diversify homecare provision and increase personal choice. Baxter et al. (2013) examined to what extent personal budgets and LA‐run brokerage service in England increased choice for homecare recipients, finding that the potential of personalisation was affected by LAs' dual objectives of (1) containing costs by reducing the number of care agencies commissioned and (2) maintaining contractual relationships with a range of stable providers. The authors found assessing the brokerage system's efficiency challenging; homecare agencies could not offer the levels of flexibility anticipated, and flexibility was impeded by LA rules that required providers to obtain permission before making even minor changes to care arrangements. They concluded that when large numbers of providers are retained on an LA's framework, care recipients are not guaranteed choice in when their care is provided, how or by whom.

Baxter et al.'s (2011) examination of the outcomes of personalisation on workforce employment found that homecare providers were expected to adjust to being employed directly by personal budget holders whilst their block contracts with the LAs were phased out. Providers faced difficulties related to finances and workforce planning when clients who purchased their service directly gave short notice of termination or used services intermittently. Providers also found care worker retention rates were minimally affected by care workers leaving to take up employment with direct payment clients; where care workers did leave, they often returned, resulting in frustration amongst providers at repeated recruitment costs.

Sutcliffe et al. (2012) argued that commissioning practices adapted for managed budgets were better able to provide flexible responses to meet the needs of individual care recipients. However, barriers to increased flexibility between localities and service providers included: lack of budgetary devolution to frontline staff; limited cash flow; limited availability of providers with an organisational culture that facilitated creative care planning.

Brookes et al. (2015) studied personal budgets and other innovative practices (e.g. integrating care with housing and preventative services), finding personalisation to be a significant driver of change (noting that the practices studied were developed pre‐austerity). They found various interventions which facilitated personalisation: resource directories and signposting; programmes to support direct payments use; an online vetting and matching service for personal assistants; assistive technology uses during assessment; funding voluntary organisations.

### 5.2 Alternative operational models

Papers in this category conceptualised alternative organisational and/or community‐based models as innovations offering more flexible, responsive and adaptive care. Jones (2009) suggested that social enterprises can create a flexible model for delivering publicly funded homecare whilst reinvesting any surplus into the organisation. The author argued that small, community‐based social enterprises tend to achieve greater workforce stability, provide more personalised care and have greater flexibility in developing and delivering services than traditional models. However, evidence of such outcomes was not presented.

Munoz (2013) evaluated the development of a social enterprise that co‐produced care services in rural Scotland. The author illustrated and discussed several difficulties in realising this project, including competing views about service development and problems balancing volunteering activities and paid employment. She also identified reticence amongst those involved to take up leadership roles within the venture and found members of the community tended to envision a traditional model of a homecare agency. The paper illustrated the complexity of community dynamics and the need to factor in local contexts when establishing new services. Overall, the shift from the in‐house provision by the locality to a social enterprise model was a source of concern for those involved, raising questions about who was ultimately responsible for provision.

Westwood's (2016) review paper identified that most independent homecare failed to address the needs of Lesbian Gay Bisexual and Trans (LGBT) individuals. The author argued for a human rights approach to developing innovative provision, reflecting the diversity within society to better meet the needs of people marginalised within the care system. Westwood underlined contradictions between what activists have called for and research findings, such as separate or integrated provision, also stressing the lack of diversity within ASC staff, and a need for a greater understanding of different people's care needs.

Cameron et al.'s (2020) paper examined if an extra‐care housing (ECH) model^v^ could support independent living and ameliorate the effects of social isolation, poor social care outcomes and high costs of residential care. Although this model of care is promoted as being more flexible, responsive and adaptable to older people's needs, the authors highlighted that in practice, the way homecare services were organised was not innovative and mirrored standard models of homecare (Bottery, 2018). Similarly, Evans et al. (2020) presented a longitudinal study of an extra‐care model for older adults with and without dementia. They argue that ECH can offer opportunities for social interaction and meaningful activity however these opportunities were not always equally accessible to all as residents with dementia felt lonely, which may be linked to the stigma and prejudice that continues to be associated with the condition.

Glasby et al.’s (2018) study compared well‐being outcomes for care recipients with the size of the homecare organisations that provided their care. Findings showed that micro‐providers (<5 paid/unpaid staff) were able to deliver more personalised care, achieve better well‐being outcomes for care recipients, and offer better value for money than larger providers. They suggested that a micro‐provider model can generate continuity in staffing, enhance worker autonomy and build care relationships between care workers and care recipients. However, micro‐providers were prone to business instability and failure because of difficulty securing a stable income. The authors concluded that expansion of direct payments could increase demand for micro‐enterprises, although small company owners/leaders experienced difficulties in gaining admittance to ‘approved provider lists' to access LA‐funded work, reducing demand for their services.

Giraud et al. (2014) analysed three innovative service models: a reablement project that aimed to increase care recipients' independence following hospitalisation; a case‐management approach; and the creation of a group lunch project for people in receipt of meals‐on‐wheels. The authors suggest that innovations are often inspired by a libertarian (democratic participation) critique of the traditional welfare state, but move to more neoliberal ideals (consumerist participation) as their services become established, expand and scale‐up.

### 5.3 Training regulation catalysing change

Rainbird et al. (2011) was the only paper found that examined the innovative potential of training regulation. The authors focused on regulations introduced through the Care Standards Act (2000) on the provision of UK care services and examined how these mechanisms triggered innovation by creating the conditions through which homecare organisations were able to comply with requirements and innovate workforce training. They argued that regulation is sometimes the catalyst to alter organisational behaviour and provide impetus to change which was ‘already going in the same direction’ (Rainbird et al., 2011. p. 3739). The example offered examined how ‘dementia training’ developed team‐working at homecare to provide more flexible and responsive care and offer an alternative to ‘the normal time‐limited and task‐oriented delivery of domiciliary care services’ (Rainbird et al., 2011: p. 3736). They note the importance of ‘multiple sources of funding and the activities of enthusiastic local actors’ (Rainbird et al., 2011. p. 3739) to foster innovative activities.

### 5.4 Assistive technology for enhancing care

Whilst the focus of recent policy and funding opportunities has been on technologies more broadly, these papers focused specifically on assistive technologies (AT). The papers in this category tended to view AT tools as innovation, and as the means to enhance homecare delivery. Caleb‐Solly's (2016) commentary piece proposed that intelligent robots, integrated with smart home sensors and healthcare databases, are important tools to enhance older people's quality of life, reduce unmet care needs and ease the effects of staff shortages in homecare. The author discussed a range of available technologies and highlighted the need to understand the low take‐up of AT, but offered weak evidence for its benefits. Doughty and Williams (2016) considered AT to have the potential to maintain and increase a person's ability to live independently at home, but also highlighted its low take‐up. They argued that care assessors tended to lack appropriate knowledge for suggesting how AT can support people to remain living independently at home, and found that a lack of support following the initial installation led to underuse by care recipients. Roberts et al. (2015) provided a more nuanced examination of the potential of ICT telehealth technologies to assist older people living in remote and rural areas to manage chronic pain. They found health and care professionals viewed technologies as a means to increase opportunities for care recipients to connect socially and access additional information about their condition and pain management. The authors suggested that ICT technologies can supplement in‐home visits and recommend including health and care professionals in decision‐making in commissioning ICT for chronic pain management. However, care workers noted that some of their care recipients did not engage with the ICT technologies, and the authors highlighted that aspects of the ICT device design, ergonomics and infrastructure, such as unreliable broadband coverage and connectivity prevented their optimal use.

## 6 DISCUSSION

The literature on innovation and ASC more generally, we suggest, is wide‐ranging, fragmented and conceptually ‘messy’. These characteristics mirror concerns related to definitional ambiguity and under‐conceptualisation of innovation within and across disciplines and sector‐based studies. Although innovation is an important priority for UK ASC policy, research into innovation in independent homecare organisations is relatively underdeveloped.

Our thematic analysis of the papers which met our inclusion criteria produced four main categories of innovation explored in the literature, and whilst we have categorised the papers by type of innovation, there is overlap between them. Many papers addressed the extent to which a personalisation agenda was being met, either directly or indirectly. One group of papers highlighted that personalised funding as a personalisation mechanism generates flexibility (Sutcliffe et al., 2012), choice (Baxter et al., 2013), change (Brookes et al., 2015) but also implications for the homecare workforce (Baxter et al., 2011), with both positive and negative outcomes. Positive outcomes included improved engagement between provider and service users, and creating renewed partnerships (Brookes et al., 2015). The findings of these studies underline the importance of micro‐level (individual and group) and meso‐level (organisational) environments for understanding the context‐specific factors that inhibit or support innovations to achieve successful and long‐lasting outcomes. One anticipated effect of personalised funding was that the market would create more diverse provision, but as has been noted this increased ‘choice’ is not always easy to achieve (Baxter et al., 2013) or available (Westwood, 2016). The market's ability to respond to needs may also have been impacted by austerity insofar as ‘personalisation’ was focused on ‘budgets and cost‐cutting’, rather than ‘choice and control’ (Brookes et al., 2015: 91). One negative effect of personalised funding was recruitment costs for homecare organisations when care workers were ‘poached’ to directly work for care recipients (Baxter et al., 2011), and may also have fuelled the recent growth in micro‐enterprises (Glasby et al., 2018). Whilst micro‐providers have been shown to be more adaptive and flexible to meet individuals' needs and improve care outcomes, they also create more precarious employment opportunities because of their susceptibility to cash‐flow problems and business fragility.

The group of papers which characterised innovation as alternative organisational models also reflected a drive for more personalised care. The review and commentary by Westwood (2016) call for greater diversity in the provision and the care workforce as well as training. Other papers explored how organisational model and size can, positively and negatively, affect working conditions, management and the sustainability of services. One outcome derived from the model of extra‐care housing was shown on the one hand to prevent social isolation (Cameron et al., 2020), but on the other hand, it can increase social isolation for residents with dementia, as the stigma from other residents without dementia can cause segregation (Evans et al., 2020). Social enterprises were claimed to have the potential to empower employees (Jones, 2009; Munoz, 2013). The evidence offered by Jones (2009) was weak, but Munoz's (2013) more robust analysis of the positive and negative effects of implementing social enterprises demonstrated that a bottom‐up approach to developing co‐produced services could foster a sense of community, empowerment and personal satisfaction. However, this also led to feelings of pressure, strain and frustration and a reluctance to embrace ‘transformative’ co‐production. The result was that the traditional provider‐user dynamics were maintained and highlights the difficulties in implementing and maintaining an ideal. Similarly, Giraud et al.'s (2014) discussion of three initiatives founded on values‐based service innovation had to adapt to the requirements and expectations of the wider social care system, demonstrating how principles driving the innovation can drift towards a more neoliberal agenda concerned with issues of income and capital.

The impact of top‐down influences on innovation and outcomes was demonstrated by Rainbird et al. (2011), who focused on the implementation of training regulation as a means to promote quality care. Central to this paper's argument is that the organisations' responses to top‐down regulation can create opportunities for innovation and that this is more likely when the intended change is aligned with the regulation's direction of travel. The focus of this paper, whilst highlighting some of the positive outcomes of innovation such as increased staff motivation and a ‘learning culture’, is about the conditions which foster innovation, with key enthusiastic personnel and funding emphasised as vital for an innovative culture to develop and nurture change.

Given the focus on technology as a policy priority (Wright, 2020), surprisingly only three papers were identified in this group. This is not to say that research around technology and care is not being undertaken, but that technology is little discussed in relation to homecare organisations, and where studies have been conducted, they have focused narrowly on assistive technology (AT). What was clear from these papers, however, is that the use of AT to promote independent living is assumed to be positive and yet the evidence to support such claims was not strong. Therefore, statements related to the positive outcomes of AT were speculative at best (Caleb‐Solly, 2016), or based on an implicit assumption that increased take‐up of such technologies would improve care outcomes (Doughty & Williams, 2016). Roberts et al.'s (2015) paper highlighted problems with poor digital infrastructure and connectivity but suggested that increased engagement with technologies will only complement, not replace, care provided by homecare organisations. A key benefit argued in the policy discourse of AT is cost‐effectiveness, but the papers did not detail the financial implications of using AT or their potential/actual savings. Whilst it was acknowledged that AT cannot replace all the features and benefits of costlier ‘hands on’, face‐to‐face care, it is argued to be very difficult to compare technology‐produced cost savings when face‐to‐face care visits provide an unquantifiable source of social support for care recipients and their families (Roberts et al., 2015). Overall, the negative outcomes of the use of technology tended to be underexplored in these papers and the benefits for those receiving and providing care were often assumed. Furthermore, AT was treated as the innovation, rather than considering if the way the AT was used in homecare was innovating the services. Positioning technology as the innovation is argued to ignore the context within which AT is located and the importance of the interactions with users in mediating outcomes. As Science and Technology Studies have highlighted, technology both shapes, as it makes some things possible or impossible, and is shaped by context and interactions (people can engage with, and can disable/enable its functions); ATs are ‘not neutral, predictable phenomena whose outcomes could easily be captured in isolation’ (Hamblin et al., 2017: p. 79).

What is apparent from our analysis is that research examining the process and operationalisation of innovation in independent homecare organisations—from initiation to implementation and diffusion – is so far underdeveloped. Whilst the research included in our review illuminated some of the positive and negative outcomes of innovation, it is equally important to build an understanding of the processes that support independent homecare to develop and change. Careful detailing to describe the focus of the innovation and the associated organisational context within which innovations are developing are necessary for building further knowledge, understanding and wider learning.

## 7 LIMITATIONS

Although we initially identified 72 papers (after removing duplicates) the number of papers finally selected for analysis is only 15. The filtering we performed led to the exclusion of several papers where care in the home was a focus but it was not clear if the homecare organisation was implementing an innovation; or where innovation was researched but it was unclear whether the care was being delivered by independent homecare organisations. There, therefore, could have been insightful studies excluded from our review. Methodologically there may be an element of subjectivity whilst identifying the type of innovation. Whilst interrater agreement offered fairly robust groupings of types of innovation, there may be the scope of subjective interpretation in the process. We did also find that the boundaries between the groupings overlapped as discussed above. The inductive and iterative approach methodologically helped guide us through this process.

## 8 CONCLUSION

Our aim was to provide a deeper insight and add nuance to how innovation in UK independent homecare organisations can be understood, identifying the form innovation can take and any corresponding outcomes to arise from the change. We identified and discussed four types of innovation: personalised funding, operational models, workforce training and AT. Viewed through the notion of innovation suggested by Singh and Aggarwal (2021), we discussed earlier in the introductory section, these findings help in building an understanding of ‘new adaptive solutions’ considered and/or implemented at the level of homecare organisations in the UK, ‘the motives’ for their use’, and in some cases, the ‘value’ these might add. Understanding innovation in homecare organisations therefore might require attention to what the new adaptive solution is (an approach to funding and/or care philosophy, operational model and/or ethos, tools or processes etc.) and why it is being implemented (cost saving, effectiveness, quality improvement in care, care jobs, to the local community).

What was less evident from the review is a focus on the organisational practices and process through which innovation is generated, achieved and sustained is largely missing. Whilst Gripenberg et al. (2012) warn of a ‘pro‐innovation bias’ in research, the findings suggest that investigation into innovation in homecare organisations examines both negative and positive outcomes (with studies of AT being the exception). Specifically, this review highlights how innovations can enrich care work jobs (Jones, 2009; Munoz, 2013), increase organisational responsiveness and flexibility (Cameron et al., 2020), promote choice (Baxter et al., 2013), but also generate unintended consequences such as the challenge of recruiting a diverse workforce (Westwood, 2016) and increasing people's dissatisfaction with care when new technologies were introduced (Doughty & Williams, 2016). Investigations of when innovation goes wrong or does not deliver on objectives were rare, although one paper (Munoz, 2013) highlighted many implementation difficulties.

To further elaborate an understanding of innovation in ASC research in general, and independent homecare organisations in particular, future research could examine the source of innovation; how innovations develop and are sustained within organisations; why some innovations work and others fail, and within which particular contexts. This would not only address a gap in existing research but also generate insight on the type of resources and form of support that could usefully assist homecare organisations in innovating adult social care services.

## AUTHOR CONTRIBUTION

Diane Burns, Kate Hamblin and Cate Goodlad contributed to the conceptualization of the study and methodological approach. Cate Goodlad and Karla Zimple‐Leal applied the methodology, including collaborating on the literature searches and review of the papers. Diane Burns prepared the initial draft. All authors contributed to the review and editing.

## PAPERS INCLUDED IN THIS REVIEW

Baxter, K., Rabiee, P., & Glendinning, C. (2013). Managed personal budgets for older people: what are English local authorities doing to facilitate personalized and flexible care? *Public Money & Management*, **33(**6), 399‐406.

Baxter, K., Wilberforce, M., & Glendinning, C. (2011). Personal Budgets and the Workforce Implications for Social Care. *Social Policy and Society*, **10**(1), 55‐65.

Brookes, N., Callaghan, Li., Netten, A., & Fox, D. (2015). Personalisation and Innovation in a Cold Financial Climate. *British Journal of Social Work*, **45**(1), 86‐103.

Caleb‐Solly, P. (2016). A brief introduction to… Assistive robotics for independent living. *Perspectives in Public Health*, **136(**2), 100‐107.

Cameron, A., Johnson, E.K., Evans, S., Lloyd, L., Darton, R., Smith, R., Porteus, J., & Atkinson, T. (2020). ‘You have got to stick to your times’: Care workers and managers’ experiences of working in extra‐care housing. *Health and Social Care in the Community*, **28**, 296‐403.

Doughty, K. & Williams, G. (2016). New models of assessment and prescription of smart assisted living technologies for personalised support of older and disabled people. *Journal of Assistive Technologies*, **10**(1), 39‐50.

Evans, S. C., Atkinson, T., Cameron, A., Johnson, E. K., Smith, R., Darton, R., Porteus, J., & Lloyd, L. (2020). Can extra care housing support the changing needs of older people living with dementia? *Dementia*, **19**, 1492‐1508.

Giraud, O., Lucas, B., Falk, K., Kumpers, S., & Lechevalier, A. (2014). Innovations in local domiciliary long‐term care: From libertarian criticism to normalisation. *Social Policy and Society*, **13**(3), 433‐444.

Glasby, J., Needham, C., Allen, K., Hall, K., & McKay, S. (2018). The Goldilocks question: what size is just right for social care providers? *International Journal of Care and Caring*, **2**(1), 65‐87.

Jones, C. (2009). How Social Enterprises can make a difference in caring for older people. *Working with Older People*, **13**(2),13‐16.

Munoz, S‐A. (2013). Co‐producing care services in rural areas: Managing Community Care. *Journal of Integrated Care*, **21**(5), 276‐287.

Rainbird, H., Leeson, E., & Munro, A. (2011). Is regulation good for skill development? Mediating actors and workplace practice in adult social care in England. *The International Journal of Human Resource Management*, **22**(18), 3727‐3741.

Roberts, A., Philip, L., Currie, M., & Mort, A. (2015). Striking a balance between in‐person care and the use of eHealth to support the older rural population with chronic pain. *International Journal of Qualitative Studies on Health and Well‐Being*. 10 10.3402/qhw.v10.27536.

Sutcliffe, C., Hughes, J., Xie, C., Chester, H., & Challis, D. (2012). Social Care in older people's services – Facilitating the flexible use of resources. *Care Management Journals*, **13**, 100‐107.

Westwood, S. (2016). LGBT ageing in the UK: spatial inequalities in older age housing/care provision. *The Journal of Poverty and Social Justice*, **24**(1), 63‐76.

## Data Availability

Not applicable.

## Endnotes

^i^
‘Locality’ is used to encompass the different terms for local government.

^ii^
Direct payments refer to cash payments from localities that allow individuals to arrange and pay directly for their own care.

^iii^
Individual budgets are a personal budget that is means and needs assessed, paid by the locality towards social care and support needs.

^iv^
Other wildcards were trialled, including ‘home care’ but these generated results which were not relevant to our research questions e.g. nursing homes, care homes and residential care.

^v^
Housing for older adults that support independent living in self‐contained accommodation with 24‐hour on‐site care.
